# Gene Expression and Epigenetic Regulation in the Prefrontal Cortex of Schizophrenia

**DOI:** 10.3390/genes14020243

**Published:** 2023-01-18

**Authors:** Wiktor Bilecki, Marzena Maćkowiak

**Affiliations:** Maj Institute of Pharmacology, Polish Academy of Sciences, Department of Pharmacology, Laboratory of Pharmacology and Brain Biostructure, Smętna Str. 12, 31-343 Kraków, Poland

**Keywords:** schizophrenia, transcription, epigenetics

## Abstract

Schizophrenia pathogenesis remains challenging to define; however, there is strong evidence that the interaction of genetic and environmental factors causes the disorder. This paper focuses on transcriptional abnormalities in the prefrontal cortex (PFC), a key anatomical structure that determines functional outcomes in schizophrenia. This review summarises genetic and epigenetic data from human studies to understand the etiological and clinical heterogeneity of schizophrenia. Gene expression studies using microarray and sequencing technologies reported the aberrant transcription of numerous genes in the PFC in patients with schizophrenia. Altered gene expression in schizophrenia is related to several biological pathways and networks (synaptic function, neurotransmission, signalling, myelination, immune/inflammatory mechanisms, energy production and response to oxidative stress). Studies investigating mechanisms driving these transcriptional abnormalities focused on alternations in transcription factors, gene promoter elements, DNA methylation, posttranslational histone modifications or posttranscriptional regulation of gene expression mediated by non-coding RNAs.

## 1. Prefrontal Cortex Development and Function

The prefrontal cortex (PFC) is the cortical region located in the anterior part of the frontal lobe. Anatomical definitions of the PFC are broadly diversified across species, accounting for various cognitive abilities [1]. Structural and functional homology is used for brain area classifications across species. Primate PFC can be divided into lateral (dorsal and ventral regions), orbitofrontal, medial and cingulate areas. Two major types of neurons create the local circuitry in the PFC: excitatory and inhibitory ones. Excitatory neurons are 70–75% of the neuronal population and use glutamate as the neurotransmitter, while 25–30% are interneurons using γ-aminobutyric acid (GABA) as the neurotransmitter [2]. Interneurons make synapses on definite compartments of pyramidal (excitatory) neurons, and they are divided into subgroups with specific neurochemical, anatomical, physiological and functional characteristics. PFC interneurons have been classified as calcium-binding proteins - parvalbumin (PV) or neuropeptides - somatostatin (SST), neuropeptide Y (NPY), vasoactive intestinal peptide (VIP) and cholecystokinin (CCK) [3]. The anatomical connections of long-range inputs formed on prefrontal GABA-ergic interneurons are mediated by stimulation of PV and/or SST expressing cells [4]

PV cells are fast-spiking non-adapting neurons, which are divided into basket cells targeting the soma and proximal dendrites of pyramidal cells and chandelier cells targeting the initial axon segment of pyramidal neurons. PV-positive cells are involved in the regulation of optimal excitatory/inhibitory (E/I) balance in the PFC. Inhibitory GABA signalling among PV-positive basket cells and excitatory pyramidal neurons is essential for proper γ oscillatory activity of neurons participating in cognitive phenomena such as working memory and attention [5,6]. Another class of interneurons in the PFC, expressing calcium-binding proteins, is composed of regular-spiking, calbindin or calretinin-positive cells targeting distal dendrites of pyramidal neurons layers I and IV of the PFC. Calretinin is localized in SST, NPY or VIP-expressing interneurons. VIP interneurons make inhibitory synapses on SST or PV interneurons [7]. SST neurons target distal dendrites of pyramidal cells in layers II, V and VI [2]. The PFC forms the administrative centre where information is processed and integrated to execute complex functions, such as planning, working memory, attention, decision making and goal-directed behaviour. This region also involves emotional processing, including affection, emotion, and social behaviour [2].

There are two vulnerable periods in PFC development, the perinatal period and adolescence, and impairments of these developmental stages result in abnormal cortical development and functional disability [8]. During the perinatal period, several critical processes for cortical growth are observed: neuronal proliferation, neuronal differentiation and synaptogenesis. In adolescence only, synapse pruning, together with increased myelinisation, is fundamental for neurodevelopment to strengthen and optimise salient connections in the adult brain. The PFC is a brain structure that sustains structural and functional development crosswise from adolescence into early adulthood. The above development trajectory corresponds with a transition in behaviour and cognitive function, i.e., gradual stabilisation of emotional reactivity, novelty seeking, cognitive control and decision making [9].

During adolescent neurodevelopment, 30% of synapses formed during childhood are lost [10]. The eliminated synapses are principally excitatory, and inhibitory neurons’ maturation depends on the excitatory neurons’ inputs. The process is necessary for establishing proper E/I balance in the adult cortex, which is essential for the network dynamics underlying cognitive processes. The PFC goes through a decline in grey matter volume, white content enlargement, and modifications in circuity cytoarchitecture (i.e., axon myelinisation), dendritic morphology and synaptic density that shape the brain and establish proper behavioural responses [11].

Dysfunction of the PFC is a dominant aspect of several psychiatric disorders, such as attention deficit hyperactivity disorder (ADHD), post-traumatic stress disorder (PTSD), bipolar disorder, anxiety and schizophrenia.

## 2. Prefrontal Cortical Pathology in Schizophrenia

Disturbances in cognitive function are crucial symptoms of schizophrenia. Impairments in attention, memory, and executive functions, i.e., the ability to plan, initiate and regulate goal-directed behaviour are present throughout the entire course of the illness. Cognitive dysfunctions are particularly difficult to treat with antipsychotic medicaments [12]. Cognitive functions, especially working memory, are associated with activating prefrontal cortex circuity, especially the dorsolateral prefrontal cortex, where several specific anatomical and neurochemical abnormalities are observed in schizophrenia [13,14].

Anatomical studies showed that the total number of neurons is not altered in schizophrenia; however, neuronal density increases, which reflects a reduction in the neuropil. These observations consisted of findings showing shorter dendritic length and a lower density of dendritic spines on pyramidal neurons, and a lower level of synaptophysin, a marker of axon terminals [15,16,17]. Moreover, a lower density of perineuronal nets (PNNs), extracellular structures stabilising synapses, support synaptic loss or destabilisation in the PFC of schizophrenia subjects [18]. Some findings indicate 60% synapse loss in schizophrenia [10].

The above alterations suggest dysfunction in neurotransmissions, especially excitatory ones. Abnormalities in glutamatergic signalling seem related to dysfunction in *N*-methyl-D-aspartate (NMDA) and α-amino-3-hydroxy-5-methyl-4-isoxazolepropionic acid (AMPA) receptors localised on dendritic spines [19]. Moreover, proton magnetic resonance spectroscopy (MRS) studies from the PFC showed reduced glutamate levels in schizophrenia [20].

Abnormalities in GABA signalling in the PFC of patients with schizophrenia were also reported. MRS studies from PFC showed mixed results with higher, lower or unchanged levels of GABA in subjects with schizophrenia. Disturbances in signalling between PV basket cells and excitatory pyramidal cells in the PFC contribute to altered γ oscillations and working memory in schizophrenia [5,6].

The PFC’s activity of pyramidal cells and interneurons is modulated by inputs from dopamine neurons located in the ventral mesencephalon. Several findings indicate a reduction in dopamine signalling in the PFC in schizophrenia, and a decrease in dopamine innervations was observed [21]. A positron emission tomography study showed an increased density of dopamine D1 receptors in subjects with schizophrenia that correlated with poor working memory that might be a secondary, possibly a neurodevelopmental, deficit in dopamine innervation [22]. Pathological changes in the PFC suggest loss of synapses and neurotransmission impairments (GABAergic, glutamatergic or dopaminergic) in schizophrenia.

## 3. Genetic Background of Schizophrenia

Schizophrenia aetiology has a strong genetic component. Gene-associated studies showed a possible relationship between some variants in human genes and the risk of schizophrenia. Some studies pointed out some candidate genes related to dopamine signalling, such as catechol O-methyltransferase (COMT) [23], monoamine oxidase (MAO), dopamine transporter (SLC6A3), and dystrobrevin-binding protein 1 (DTNBP1) [24]. COMT, the dopamine-degrading enzyme, is a main regulator of the prefrontal dopamine level. The allelic variants (Val158Met) in the COMT gene code slightly increase the risk of schizophrenia with increasing prefrontal dopamine catabolism and impairing prefrontal cognition [25]. MAO is a mitochondrial enzyme existing in two forms, MAO-A and MAO-B. MAO plays an essential role in dopamine degradation and regulation of dopaminergic neuron activity. The MAO-B rs 1799836 polymorphism (A to G substitution) was suggested to be connected with the aetiology of schizophrenia and negative symptoms development [25]. Dysbindin, a protein encoded by DTNBP1, is located in the synapses. Dysbindin C-A-T haplotype (risk allele of DTNBP1 rs2619539, rs3213207, rs2619538) is associated with increased risk of schizophrenia [26] and affects brain structure reducing grey matter volume [27].

Key loci associated with schizophrenia risk are related to excitatory neurotransmission: the NMDA receptor (subunit 2A; *GRIN2A,* the estimated odds ratio for highly damaging coding variants ~24.1), AMPA receptor subunit (*GRIA3*; the estimated odds ratio for highly damaging coding variants ~20.1) as well as various postsynaptic cell adhesion and scaffolding proteins of excitatory synapses like postsynaptic density protein 93 (PSD-93) and synaptic Ras GTPase activating protein (SYNGAP1), which regulate the NMDA receptor-dependent trafficking of AMPA receptors and synaptic potentiation and are required for proper synaptic function [28,29]. Genetic advances show that schizophrenia is also associated with variants linked to genes affecting GABAergic transmission. Schizophrenia-associated loci encoding proteins involved in inhibitory neurotransmission include GABAB receptor components GABBR1 and GABBR2 and loci linked to proteins that mediate GABA receptor turnover such as ankyrin-G (ANK3), which stabilises somatodendritic GABAergic synapses and, in an rs41283526 variant could be protective against schizophrenia. Furin, a protein affecting inhibitory synaptic transmission by altering the transcription of GABAA receptor subunits, has been implicated in schizophrenia GWASs along with chloride channel *CLCN3* and vesicular inhibitory amino acid transporter *SLC32A1*, involved in GABA uptake into synaptic vesicles [28,30,31].

A genome-wide associated study of more than 36,000 schizophrenia patients and 100,000 controls identified 128 independent associations in 108 loci. Noted associations were relevant to dopamine D2 receptors and many genes involved in glutamatergic neurotransmission and synaptic plasticity (glutamate metabotropic receptor 3, GRM3; glutamate ionotropic receptor NMDA type subunit 2A, GRIN2A; serine racemase, SRR; glutamate ionotropic receptor AMPA type subunit 1, GRIA1). In addition, associations at calcium voltage-gated channel subunit α 1C (CACNA1C), calcium voltage-gated channel auxiliary subunit β 2 (CACNB2) and calcium voltage-gated channel subunit α 1 I (CACNA1I), which encode voltage-gated calcium channel subunits, were also reported [32]. Studies of rare genetic variations also showed genes encoding calcium channels, proteins involved in glutamatergic transmission and synaptic plasticity as a schizophrenia risk [33,34,35]. Moreover, risk variants for schizophrenia aggregate in specific biological pathways such as postsynaptic density, postsynaptic membrane, dendritic spine, and axon part [36]. A recent study using single-cell RNA-sequencing showed in the human-specific granular layer of the PFC 6 schizophrenia-related genes associated with synaptic transmission, cell development and differentiation (Met proto-oncogene, receptor tyrosine kinase, MET; Neurogranin, NRGN; parvalbumin, PVALB; retinoic acid receptor β, RARB; thymocyte expressed, positive selection associated 1, TESPA1; and zinc finger matrin-type 4, ZMAT4). Some of these genes (RARB, ZMAT4) were correlated with grey matter volume differences between patients with schizophrenia and healthy control [37]. Thus, the genetic predisposition to schizophrenia might be related to the regulation of synaptic neurotransmission (i.e., dopaminergic, glutaminergic) and synaptic plasticity.

## 4. Abnormalities in Cortical Gene Expression in Schizophrenia

Gene expression studies using several methods, including RT-PCR technique, microarray technologies and transcriptome sequencing, showed alterations in gene transcription in the PFC of patients with schizophrenia. Several theories have been proposed for the pathophysiology of psychosis. Dopamine and glutamate theories are the most popular, and the immune system and energy metabolism dysfunctions are the most recent [38]. The dopamine theory implies that dopamine hyperactivity at the D2 receptors of the dopaminergic pathway causes positive symptoms. This observation is further supported by the fact that all antipsychotics are aimed at the blockade of D2 receptors. The glutamate theory suggests that the NMDA receptor is less functional in the prefrontal cortex. The neuroinflammatory idea has linked microglia activation to cognitive decline, while brain energy theory suggests that energy metabolism is compromised in schizophrenia.

### 4.1. Glutamate-Related Genes

Postmortem studies of schizophrenia subjects indicated that changes in mRNA or protein level of NMDA receptor subunits in the PFC were relatively minor and not consistently replicable [19]. A decrease in GRIN1 and GRIN2C mRNA were observed, but no changes in GRN2A and GRIN2B were detected in the PFC of schizophrenia subjects [39]. In some analyses, GRIN1, GRIN2A and GRIN2B were unchanged, but GRIN2A vs. GRIN2B ratio decreased in the PFC of patients with schizophrenia [40]. Another study reported less GRIN1, GRIN2A and GRIN2C mRNA in the lack of alterations in GRIN2B and GRIN2D mRNA levels [41]. Significant different expression of GRIN3A was observed in the PFC patients with schizophrenia compared to healthy subjects [42].

Some studies did not detect changes in the mRNA level of NMDA receptor subunits in the PFC of schizophrenia subjects [43]. However, a decrease in mRNA of postsynaptic density 95 protein (PSD95) binding NMDA receptor subunits was reported in the PFC of subjects with schizophrenia [44], but no changes were also reported [41].

In the case of AMPA receptors, findings from postmortem studies are also inconsistent, showing an increase in expression of some subunits of AMPA receptor, especially in GRIA1 and glutamate ionotropic receptor AMPA type subunit 4 (GRIA4), reporting a decrease in glutamate ionotropic receptor AMPA type subunit 2 (GRIA2) or no significant differences [45,46].

In the case of metabotropic glutamate receptors, a higher mRNA level for mGluR1α was detected in the PFC of schizophrenia subjects [47]. A similar increase in mRNA level was observed for mGluR2 but not for mGluR3 [48].

The mRNA levels of excitatory amino acid transporters, i.e., EAAT1 and EAAT2, were also analysed in the cortical areas of schizophrenia subjected. The findings showed an increase in mRNA of EAAT1 [49,50,51] or an unaffected mRNA in EAA1 transcripts in patients with schizophrenia [52]. The expression of EAAT2 has also increased [50], but no changes in the EAAT2 mRNA were also reported in patients with schizophrenia [49,52].

### 4.2. GABA-Related Genes

Postmortem study of schizophrenia subjects showed reduced levels of mRNA of glutamic acid decarboxylase 67 (GAD67), a principle synthesising enzyme for GABA, and mRNA of PV and SST proteins in the PFC [53,54]. Some findings also reported changes in the presynaptic side of GABA synapses, such as a decrease in mRNA levels of GABA transporter 1 (GAT1), a GABA membrane transporter responsible for the reuptake of released GABA into nerve terminals [55] or mRNA levels of the vesicular GABA transporter (vGAT) that loads GABA into presynaptic vesicles [53] in the PFC of schizophrenia subjects. On the postsynaptic side, mRNA levels of α1 and α2 subunits of the GABAA receptor (GABRA1 and GABRA2) were lower and higher, respectively, in some studies of schizophrenia [53,56]. Significant differences in the expression of genes encoding α5 and β3 subunits of GABAAreceptor (GABRA5 and GABRB3) were also detected in the PFC of patients with schizophrenia [42].

### 4.3. Dopamine-Related Genes

The level of COMT mRNA and protein does not appear to be altered in schizophrenia [57]. Findings analysing the mRNA level of COMT showed no difference in terms of the mean level of COMT in healthy controls and schizophrenia subjects; however, there was a significant difference in the laminar pattern of COMT mRNA in pyramidal neurons. The expression of COMT mRNA was homogenous across cortical layers, whereas patients with schizophrenia had a lower level in the superficial layers and higher in the intermediate/deep layers [58].

Dopamine receptor studies showed a decrease in mRNA of the D1 receptor in the PFC of schizophrenia subjects [59], and a reduction of the prefrontal D1 receptor in patients with schizophrenia was confirmed by positron emission tomography (PET) studies [60]. In the case of the D2 receptor, some results revealed a decrease [54,59] or increase in D2 receptor expression [59] in the PFC of subjects with schizophrenia [54]. However, additional findings did not show changes in D2 mRNA levels in the PFC of schizophrenia subjects [61]. Another study reported a decrease in D3 and D4 receptor transcripts in the PFC of schizophrenia patients [62].

### 4.4. Plasticity-Related Genes

Early studies showed a decrease in the mRNA level of reelin, an extracellular glycoprotein secreted by GABA-ergic interneurons involved in dendritic plasticity [54]. A reduction in mRNA level of DTNBP1 gene-modified synapse function was reported in the human cortex [63]. Some evidence showed differences in the expression of genes encoding subunits of ion channels in the PFC of patients with schizophrenia compared to healthy subjects. Significant different expression was observed in voltage-gated ion channels (potassium voltage-gated channel subfamily A member 1, KCNA1; potassium voltage-gated channel subfamily C member 3, KCNC3; potassium two pore domain channel subfamily K member 1, KCNK1; sodium voltage-gated channel α subunit t9, SCN9A), transporters (solute carrier family 16 member 2, SLC16A2; solute carrier family 25 member 33, SLC25A33; solute carrier family 26 member 11, SLC26A11; solute carrier family 35 member F2, SLC35F2; solute carrier family 7 member 3, SLC7A3) and ion channel auxiliary subunits (potassium voltage-gated channel interacting protein 3, KCNIP3; sodium voltage-gated channel β subunit 1, SCN1B) [42]. Other studies showed the increased transcript of genes involved in synaptic plasticity: calcyon, a protein potentiating crosstalk between the D1 dopamine receptor and G_q-11_-links receptor, and spinophilin, a protein enriched in dendritic spines and modulating excitatory neurotransmission [64], and also downregulation of synaptogyrin 1 (SYNGR1) and synaptogamin (SYT11) regulated presynaptic plasticity the PFC of patients with schizophrenia [65]. Moreover, in patients with schizophrenia, a reduction in gene expression related to cytoskeletal modification, such as growth-associated protein 43 (GAP43) and neuronal navigators (NAV1), was reported [66]. Some findings also indicate a decrease in the expression of genes related to growth factor pathways in the PFC of patients with schizophrenia, i.e., vascular endothelial growth factor receptor 2 (VEGFR2/KDR) [67]; Sprouty 2, a regulator of growth factor signalling; and brain-derived neurotrophic factor (BDNF) [68].

### 4.5. Myelination-Related Genes

Some studies showed a decrease in the mRNA level of oligodendrocyte-specific transcription factor, sex-determining region Y-box containing gene (SOX10) in the diseased PFC, [69]. Genes enriched in myelin-forming oligodendrocytes were also transcriptionally downregulated in the PFC in schizophrenia subjects: myelin and lymphocyte protein (MAL), 2′,3′-cyclic nuclei 3′-phosphodiestarase, myelin-associated protein (MAG), transferrin, gelsolin, HER3 (ErbB3) [70]. Moreover, the downregulation of myelin-related and oligodendrocyte genes such as proteolipid protein 1 (PLP1), transferrin (TF), oligodendrocyte transcription factor 1 (OLIG1), and upregulation of myelin basic protein (MBP) were reported in the PFC of patients with schizophrenia [71].

### 4.6. Metabolic-Related Genes

A cDNA microarray study showed a reduction in metabolic gene expression in the PFC in schizophrenia. These genes were categorised into five metabolic pathways involved in the regulation of ornithine and polyamine metabolism (antizyme inhibitor, ornithine aminotransferase), the mitochondrial malate shuttle system (translocase of inner mitochondrial membrane 17), the transcarboxylic acid cycle (malate dehydrase 1, NAD), aspartate and alanine metabolism (glutamic-oxaloacetic transaminase 2, mitochondrial), and ubiquitin metabolism (ubiquitin-specific protease 14, ubiquitin C-terminal esterase L1) [72]. A reduction in ubiquitin-conjugating enzyme E2N (UBE2N) in the PFC of patients with schizophrenia was also reported [45]. The above changes were supported by another study showing the downregulation of the expression of genes involved in mitochondrial and ubiquitin-proteasome system function in the PFC [73].

Some changes in gene expression specific to glial cells were also observed in the PFC in patients with schizophrenia. An increased mRNA level of the astrocytic gene aldehyde dehydrogenase-1 family 11 (ALDH1L1) and expression of glutamine synthetase (SG) were observed [74]. Alterations in the expression of microglia-related genes were also reported: an increase in chemokine (C-X3-C motif) ligand 1 (CX3CR1) mRNA and a decrease in mRNA of receptor expressed on myeloid cells 2 (TREM2), which is involved in microglial metabolism [74].

### 4.7. Inflammation-Related Genes

Higher mRNA levels of most NF-κB family members (that provide control over cytokine production), as well as NF-κB activation receptors and NF-κB–regulated transcripts (IκBα), were observed in the PFC of schizophrenia subjects and proposed to reflect the disease process [75].

High-throughput gene expression studies using RNA from postmortem brain revealed upregulation of genes associated with immune/inflammation response and cytokine production in schizophrenia cases, with TNFRSF1A, YBX3, ANGPTL4 and PXDC1 being the top hub genes in the PFC [76].

On the other hand, genes involved in immune homeostasis, including pro- and anti-inflammatory cytokines, cytokine modulators (toll-like receptors, colony-stimulating factors, and members of the complement system, CSF1R, TLR4, IL6, TNFα, TNFRSF1A, IL10, IL10RA and IL10RB) were downregulated and correlated with microglial marker CD68 in the PFC of chronic schizophrenia patients [77]

## 5. Epigenetic Regulation

Schizophrenia is highly heritable, but the genetic background does not exclusively explain the aetiology and pathogenesis of schizophrenia. A low rate of concordance in schizophrenia symptoms in identical twins indicates that other factors besides genetics are involved in schizophrenia development [78]. Several findings indicate that the vulnerability factors for the development of schizophrenia in later life include pregnancy, childhood trauma, intoxication, and sleep deprivation. All identified factors might interact with a genetic pattern of an individual, leading to manifestations of various phenotypes during development [79]. Such gene-environment interactions are associated with epigenetics. Epigenetics mechanisms mediate environmental to gene expression without directly affecting DNA sequence. Chromatin can exist in different states, including open (eu) and condensed (hetero) chromatin. Three characteristics differentially define these: loose or dense nuclear packaging, specific types of posttranslational histone modifications, and the presence or absence of various chromatin regulatory proteins that either facilitate or repress transcription. Apart from the linear genome in two-dimensional regulation, the spatial configuration and packaging of interphase chromosomes (the three-dimensional (3D) genome structures) are considered to be essential for gene expression regulation and maintenance of genome integrity and stability [80].

Epigenomic markers associated with open (active) chromatin permissive for gene expression or repressed (silencing) chromatin suppressing gene expression are explored in the postmortem brains of subjects diagnosed with schizophrenia. Findings from candidate gene studies and genome-wide mappings of DNA methylation and histone modifications indicate that alterations in chromatin structure and function might contribute to transcriptional dysregulation in the brains of subjects with schizophrenia [80]. Epigenetic changes affect gene expression more extensively than genetic variants but, unlike genetic changes, are reversible, which makes them susceptible to pharmacological manipulation. Hence, an increasing number of small molecules, termed epigenetic drugs, are being developed to alter DNA and chromatin structure, inhibit the deposition of chromatin marks or disrupt transcriptional and posttranscriptional modifications. The possibility of reprogramming the epigenome of patients bears potential clinical implications of epigenetic therapies to prevent or reverse SCZ [81].

### 5.1. DNA Methylation

DNA methylation is one of the key epigenetic mechanisms involved in regulating gene expression. DNA methylation is catalysed by DNA methyltransferases (DNMTs: DNMT1, DNMT3A, DNMT3B and DNMT3L). DNMTs are involved in the transfer of methyl group from S-adenyl-L-methionine to the cytosine residues at the C5 position to form 5-methylcytosine (5mC) and homocysteine. 5mC is a marker of DNA methylation, whereas the hydroxylated form of 5mC (5hmC: 5-hydroxymethylcytosine, 5-formylcytosine, 5-carboxylcytosine) signifies an active demethylation process [82].

Methylation at the cytosine C5 position, primarily in the context of cytosine–guanine (CpG) dinucleotides, when located in gene promoters, often is implicated in gene repression and directly impends the binding of transcription factors and induces mainly repressive chromatin structure non-permissive for transcription [82]. Analysis of DNA methylation in cells isolated from PFC indicated a significantly higher potential to change methylation profiles in neuronal than non-neuronal cells. Moreover, the majority of differential methylation between neurons and non-neuronal cells harbours a strong signal for genetic risk for schizophrenia [83]. DNA methylation patterns detected in neuronal nuclei were distinctive and correlated with promoter function [84]. Comparison between two significant populations of prefrontal cortex neurons (GABAergic interneurons and glutamatergic neurons) showed differences in methylation in CpG, non-CpG and hydroxymethylation (hCpG). Glutamatergic neurons revealed more undermethylated CpG sites and hCpG than GABA neurons. In contrast, a comparable number of undermethylated non-CpG sites was identified in both subtypes of neurons [85].

A postmortem study showed that DNA methylation levels in the PFC are associated with gene sequencing and developmental stage. Moreover, developmentally associated changes in DNA methylation were enriched for genomic regions related to clinical risk for schizophrenia. In addition, the results showed several thousand CpGs demonstrated statistically significant differences in DNA methylation levels between adult patients with schizophrenia and healthy subjects [86]. Moreover, another finding of the DNA methylation pattern of PFC neurons indicates that regions differentially methylated in GABA and glutamatergic cells were also significantly enriched for schizophrenia risk loci [85].

Early studies examining the epigenetic status of candidate genes affected in schizophrenia showed that reelin promoter is hypermethylated in the cortex of subjects diagnosed with schizophrenia [87]. The SOX10 gene was also highly methylated in the PFC of patients with schizophrenia [69], pointing to oligodendrocyte dysfunction in schizophrenia. Genome-wide mapping of the DNA methylome in the frontal cortex of subjects with schizophrenia reported alteration in the methylation status of genes implicated in excitatory (e.g., NMDA receptor subunits, gene GRIN3B; AMPA receptor subunits, gene GRIA2) or inhibitory transmission (e.g., gene MARLIN-1, RNA-binding protein regulating GABA(B) receptor) [88]. The changes in DNA methylation of genes related to excitatory transmission, i.e., GRIA4, were also reported in the other study [89]. Other findings confirmed the changes in DNA methylation and transcription of the inhibitory transmission gene glutamic acid decarboxylase (GAD1) [90], while no differences in CpG methylation PV promoter sequence were observed in the PFC of schizophrenia subjects [91]. A decrease in the mRNA level of cannabinoid receptor 1 (CB1) in the PFC of schizophrenia subjects was associated with the DNA methylation level of the CNR1 gene-encoded CB1 receptor [92]. Another study showed modifications in the DNA methylation of genes linked to dopaminergic neurotransmission (COMT, DRD2) or DNA methylation (DNMT1) [93]. DNA methylation and gene expression studies also showed differences in PFC of schizophrenia patients and healthy controls in genes related to astrocyte-associated functional pathways and oligodendrocyte development and myelination i.e., SOX10 [93,94]. Additional genome-wide methylation array revealed 37 differentially methylated genes in schizophrenic and healthy control, among which are genes regulating transmission or metabolic processes (i.e., iron-sulfur cluster assembly factor 1, NUBP1; serine/threonine kinase 32B, STK32B; androgen induced 1, A1G1; protein kinase C epsilon, PRKCE; Ras P21 protein activator 3, RASA3; and ATPase phospholipid transporting 11A, ATP11A) [95].

Thus, DNA methylation status is affected in schizophrenia which influences gene expression in the PFC.

### 5.2. Histone Modifications

Several findings showed many histone modifications marked by specific groups (i.e., methyl, acetyl phosphate groups). Histone modification processes are complex and involve writers modifying specific substrates by adding groups (enzymes, i.e., histone methyltransferases, HMTs; histone acetyltransferase, HATs; kinases), erasers catalysing the removal of specific histone modifications (enzymes, i.e., histone demethylases (HDMs), histone deacetylases (HDACs), and readers, regulatory proteins containing unique domains that recognise specific groups (i.e., methyl, acetyl, phosphate)) [96].

#### 5.2.1. Histone Methylation

Histone methylation can exist in multiple valence states: mono (me1), di (me2) and trimethylation (me3) forms. The methylation of lysine (K) and arginine (R) residues can be associated with either gene activation or repression, depending on the residues being modified [96].

The genome-wide study identified an association between schizophrenia risk genes and histone methylation, mainly histone H3-lysine 4 (K4) methylation (H3K4me3) [36]. Moreover, epigenetic profiling with neuronal and non-neuronal cells from cortical areas of patients with schizophrenia showed that schizophrenia risk variants are enriched in neuronal H3K4me3 landscapes [97]. In addition, whole-genome CHIP-seq data obtained from chromatin regions marked by H3K4me3 in PFC neurons identified multiple loci with up and downregulated open chromatin in schizophrenia patients. The above changes corresponded to the downregulation of genes involved in inhibitory neurotransmission (GAD1) [98]. The groups of genes with dysregulation of H3K4me3 loci were enriched with genes for oxidative response (i.e., platelet-derived growth factor receptor, β polypeptide, PDGFRB) and regulation of cell motility (i.e., multimerin 2, MNRN2) [99]. On the other hand, chromatin immunoprecipitation followed by deep sequencing (ChIP-Seq.) study of trimethyl H3 (H3K4me3) methylone did not show disease-sensitive changes in the PFC neurons of schizophrenia subjects [100]. Thus, the role of H3K4me3 in the regulation of gene expression in the PFC needs to be further investigated. Especially in the context of information that the HMT, SETD1A, which governs the presence of H3K4me3 in the genome [101], is one of the mutated genes in patients with schizophrenia [102].

Across histone methylation marks, high histone H3 arginine 17 (H3R17me) methylation was detected in the PFC of subjects with schizophrenia. The above modification was related to decreased expression of four metabolic genes (malate dehydrogenase, MDH; crystallin, CRYM; ornithine aminotransferase, OAT; cytochrome c1, CYTOC/CYC1 [103].

The above findings might suggest that the histone methylation process might be one of the essential epigenetic regulators affected in schizophrenia.

#### 5.2.2. Histone Acetylation

Acetylation at several lysine residues throughout the *N*-terminal tails of core histone proteins is generally correlated with gene activation [96]. A ChIP-seq. study analysing promoter and enhancer-enriched nucleosomal histone modification landscapes from adult PFC from H3K27ac profiles showed large clusters of the hyperacetylated cis-regulatory domain (CRDs) in patients with schizophrenia. Most of these clusters were enriched with genes essential for schizophrenia heritability, with the representation of regulatory sequences controlling fetal development and glutamatergic signalling [100]. The above results might suggest that disease-associated alterations affect H3K27ac acetylome in the PFC. The other study revealed that promoter-associated H3K9K14ac levels in the PFC were correlated with some gene expressions such as GAD1, 5-hydroxytryptamine receptor 2C (HTR2C), translocase of outer mitochondrial membrane 70 homolog A (TOMM70A), protein phosphatase 1E (PPM1E). Moreover, hypoacetylation of H3K9K14 was detected in the PFC of young subjects with schizophrenia [104], which might imply an essential role of histone acetylation in schizophrenia development. The role of dysregulation in histone acetylation in patients with schizophrenia confirmed proteomic study using pluripotent stem cells derived from forebrain neurons from individuals with schizophrenia. The result showed hyperacetylation of H2A.Z and H4 in neurons, and inhibition of histone acetylation readers, bromodomain and extraterminal (BET) family proteins, improves transcriptional abnormalities in neurons of patients with schizophrenia [105].

HDACs regulate histone acetylation, and their expression was examined in the postmortem study of the PFC in patients with schizophrenia. Some findings showed an increase in HDAC1 [106], and other studies reported a decrease in HDAC2 but no change in HDAC1 expression [107]. Neuroimaging findings also revealed relatively lower HDAC expression in the PFC in schizophrenia compared with controls, and HDAC expression positively correlated with cognitive performance scores among examined groups [108].

Thus, histone acetylation in schizophrenia is affected at several levels of regulation, i.e., histone erasers and histone readers.

### 5.3. Non-Coding RNAs

Non-coding RNAs are RNAs not translated into proteins, however, they are functionally active in the regulation of gene expression [109]. Among different non-coding RNA classes, the microRNAs (miRNAs) are the most studied. They are a family of short (21–25 nucleotides) RNA sequences targeting hundreds of genes via sequence complementarily to the 3′ untranslated region of the mRNA. Consequently, miRNAs negatively regulate gene expression at the posttranscriptional level. Several findings showed that one miRNA could regulate the expression of more than one target, and a single mRNA target can be controlled by several miRNAs [110].

Several findings indicate that miRNAs are involved in the critical processes of gene expression regulation in the PFC [111]. It was reported that miR137 polygenic risk affected functional connectivity in the PFC in schizophrenia [112]. The study analysing gene-miRNA interaction network identified miRNAs (miR-92a, miR-495, miR-134) directly regulating gene expression by the binding site in BCL11 A, PLP1 and SYT11, which are changed in the PFC in patients with schizophrenia. The affected genes are involved in the development and oligodendrocyte function [65].

MicroRNA expression profiling study showed a significant schizophrenia-associated increase in global microRNA expression in the PFC. The changes were associated with an elevation of DGCR8, a key component of the microprocessor complex governing the maturation of most microRNAs. Microarray analysis identified differential expression of 28 miRNA in the schizophrenia group; most of them (89%) were upregulated (i.e., miR-328, miR-17-sp, miR134, miR-652, miR-382, and miR-107). In addition, the upregulation of Dicer, which facilitates the activation of the RNA-induced silencing complex (RISC), which is essential for RNA interference, was also observed. The increased microRNAs (mainly miR-15 family members) participate in the regulation of gene expression in pathways related to synaptic plasticity [113]. Such changes might suggest altered miRNA biogenesis, which targets genes involved in synaptic plasticity (axon guidance and long-term potentiation) [71].

## 6. Conclusions

**Figure 1:**
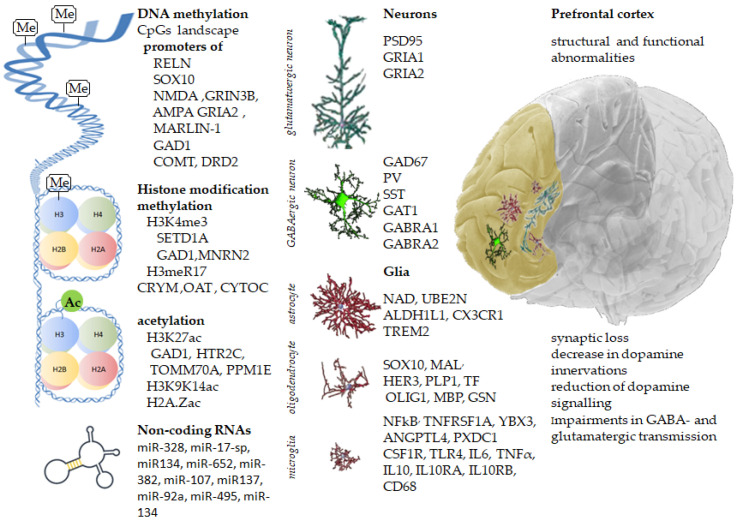
Inherited gene variants, along with acquired epigenetic modifications, change the prefrontal cortex’s molecular landscape, leading to structural and functional abnormalities in schizophrenia.

Understanding the biology of schizophrenia has been difficult because of etiological and clinical heterogeneity. Recent findings from postmortem brain studies indicate that several systems in the PFC are dysregulated due to abnormal gene expression, and alterations in gene expression in schizophrenia are linked to changes in DNA methylation and posttranslational histone modifications (Figure 1). The exact mechanisms of cortical malfunctions are still under investigation; however, several studies point to developmental risk factors, including maternal immune activation during pregnancy and upbringing conditions in adolescence, as well as drugs and toxins, to lasting epigenetic alterations in the adult brain. Finally, many of the abovementioned alterations are considered reversible and bear potential promise for novel drug targets.

## Data Availability

Not applicable.
